# Through-the-scope tack and suture system for closure of a large iatrogenic rectal perforation

**DOI:** 10.1055/a-2515-3888

**Published:** 2025-02-11

**Authors:** Lidia Marti Romero, Vanesa Martinez Escapa, Pablo Olcina Domínguez, Gloria Alemany Pérez, Carlos Boix Clemente, Belen Bardisa de la iglesia

**Affiliations:** 1 69557 Digestive Section, Hospital Francesc de Borja, Gandia, Spain; 2 69557 Department of Anesthesia, Hospital Francesc de Borja, Gandia, Spain

Early closure of an iatrogenic gastrointestinal defect reduces related adverse events. The closure method depends on the size and location of the defect 1 2 . Delayed closure may complicate treatment.

Our patient was a 68-year-old man with sepsis secondary to iatrogenic perforation of the lower rectum during a Bricker-type cystectomy for bladder carcinoma T2N0M0. During surgery, the anterior rectal wall was accidentally perforated and sutured in the same act. The patient did not recover well after the operation (clinical and lab evaluations). On the 10th postoperative day, persistent rectal perforation was confirmed by computed tomography (CT). The patient was reoperated on to suture the rectal lesion, but without success, and a loop colostomy was performed. A further 7 days later his clinical condition had worsened, with hemodynamic changes and sepsis. Repeat CT demonstrated persistence of the perforation and a large air–fluid level at the left lateroconal fascia, requiring percutaneous drainage. A colonoscopy was performed and showed abundant fecal remains in the rectal ampulla. A 35-mm orifice was observed at 3 cm from the anal margin, connecting with the peritoneal cavity with abundant purulent and fecaloid content. Inside the cavity, a surgical drainage catheter was visualized and was relocated under endoscopic vision until it drained the collection. As peritoneal communication contraindicates endoscopic placement of a vacuum system, a fully covered 20 mm × 10 cm stent (Niti-S Enteral Colonic Covered Stent; Taewoong Medical) was inserted with its distal end outside the anal margin. Ten days later, the stent was removed. The perforation edges had matured ( Fig. 1 ), so we treated them with argon and sutured them using a novel endoscopic suturing device: 4 through-the-scope tack device kits (X-Tack Endoscopic HeliX Tacking System; Boston Scientific) ( Video 1 ). Complete closure of the perforation was achieved. After this procedure, the patient had a favorable outcome, without associated rectal tenesmus. After 2 days, he was discharged.

**Fig. 1:**
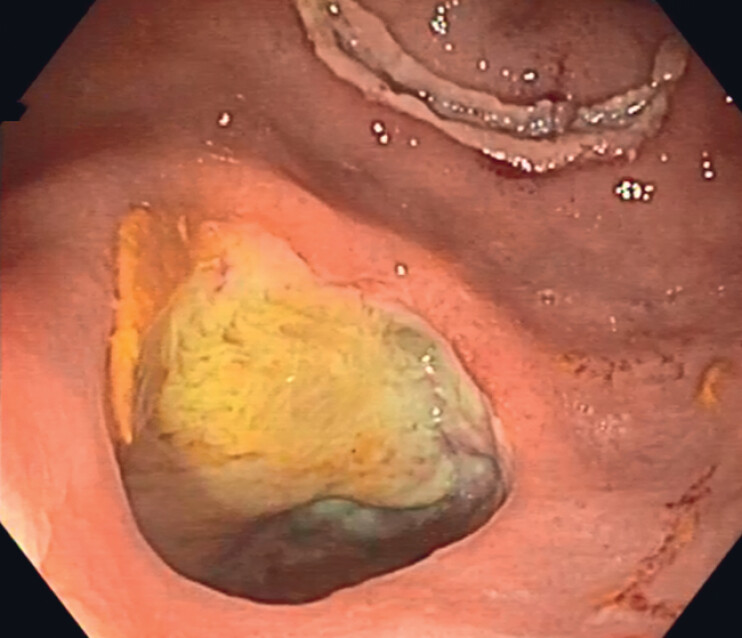
Iatrogenic rectal perforation in a 68-year-old man.

Through-the-scope tack and suture system used to repair a large iatrogenic rectal perforation.Video 1

X-Tack is a simple, useful, and accessible suture method with a low complication rate, and is a minimally invasive alternative to surgery, enabling the closure of large iatrogenic gastrointestinal perforations 3 .

Endoscopy_UCTN_Code_CPL_1AJ_2AD_3AD

## Notes

Endoscopy E-Videos https://eref.thieme.de/e-videos
*E-Videos* is an open access online section of the journal *Endoscopy* , reporting on interesting cases and new techniques in gastroenterological endoscopy. All papers include a high-quality video and are published with a Creative Commons CC-BY license. Endoscopy E-Videos qualify for HINARI discounts and waivers and eligibility is automatically checked during the submission process. We grant 100% waivers to articles whose corresponding authors are based in Group A countries and 50% waivers to those who are based in Group B countries as classified by Research4Life (see: https://www.research4life.org/access/eligibility/ ). This section has its own submission website at https://mc.manuscriptcentral.com/e-videos .

## References

[1] Canakis A Deliwala SS Frohlinger M Endoscopic outcomes using a novel through-the-scope tack and suture system for gastrointestinal defect closure: a systematic review and meta-analysis Endoscopy 2024 56 605 611 10.1055/a-2284-7334 38519045

[2] De Cristofaro E Lafeuille P Rivory J Large defect closure using a helix tacking system and endoclips after endoscopic submucosal dissection of two adjacent colonic lesions Endoscopy 2024 56 E443 E444 10.1055/a-2318-3282 38810977 PMC11136558

[3] Ebigbo A Wanzl J Afify S Endoscopic suture-based closure of a dehiscent rectal stump Endoscopy 2024 56 E381 10.1055/a-2304-8401 38684202 PMC11057958

